# Enzymic retrodifferentiation during hepatocarcinogenesis and liver regeneration in rats in vivo.

**DOI:** 10.1038/bjc.1983.222

**Published:** 1983-10

**Authors:** N. J. Curtin, K. Snell

## Abstract

The work presented here has concerned the study of early, as well as late, enzymic changes occurring during diethylnitrosamine-induced hepatocarcinogenesis and liver regeneration after partial hepatectomy in comparison with normal liver differentiation. Rank correlation analysis of the enzyme data suggested a step-wise retrodifferentiation i.e. that the liver during carcinogenesis first assumed a neonatal enzymic pattern before attaining a foetal enzymic state. Similar enzymic changes were observed in regenerating liver after partial hepatectomy; again there was a step-wise retrodifferentiation of enzymic pattern and at 3 days post hepatectomy the liver had an enzymic pattern similar to both foetal and neoplastic liver. However, in contrast to liver undergoing neoplastic change, the regenerating liver retained the capacity to undergo redifferentiation towards a normal adult biochemical pattern.


Br. J. Cancer (1983), 48, 495–505

### Enzymic retrodifferentiation during hepatocarcinogenesis and liver regeneration in rats *in vivo*

N.J. Curtin* & K. Snell

*Division of Toxicology, Department of Biochemistry, University of Surrey, Guildford, Surrey GU2 5XH*

**Summary** The work presented here has concerned the study of early, as well as late, enzymic changes occurring during diethylnitrosamine-induced hepatocarcinogenesis and liver regeneration after partial hepatectomy in comparison with normal liver differentiation. Rank correlation analysis of the enzyme data suggested a step-wise retrodifferentiation i.e. that the liver during carcinogenesis first assumed a neonatal enzymic pattern before attaining a foetal enzymic state. Similar enzymic changes were observed in regenerating liver after partial hepatectomy; again there was a step-wise retrodifferentiation of enzymic pattern and at 3 days post hepatectomy the liver had an enzymic pattern similar to both foetal and neoplastic liver. However, in contrast to liver undergoing neoplastic change, the regenerating liver retained the capacity to undergo redifferentiation towards a normal adult biochemical pattern.

A number of studies have shown neoplastic liver cells to be similar to foetal liver cells both in terms of biological appearance and behaviour (Anderson & Coggin, 1974; Farber, 1976; Enomoto *et al.*, 1978) and in terms of biochemical markers. Foetal antigens e.g., α-foetoprotein (Abelev, 1971) and isoenzymes (Criss, 1971; Schapira, 1973; Ichihara, 1975; Fishman & Singer, 1975) have been shown to reappear in liver tumours. In his work on the biochemistry of cancer Greenstein (1954) observed that "As a whole the metabolic behaviour of hepatoma and foetal liver is nearly similar and quite different from that of the nearly similar metabolic properties of resting adult liver and regenerating liver after partial hepatectomy." Collation and statistical analysis of more recent data from various sources has enabled Knox (1976) to substantiate this statement.

The resurgence of foetal properties associated with neoplastic transformation could arise in a number of ways. The process could be due to the selection of "stem cells" phenotypically similar to foetal cells which, instead of undergoing normal maturation, abnormally differentiate into malignant cells (Pierce, 1970). Alternatively, embryonic or partially specialized stem cells may be formed from adult cells by the action of the carcinogen and then blocked at any one of a number of stages of re-ontogeny (Walker & Potter, 1972). Similarly, it has been suggested that the carcinogen induces a step-wise retrodifferentiation of mature cells by a reversal of normal ontogeny (Uriel, 1976).

*Present address: Public Health Laboratory, Institute of Pathology, General Hospital, Westage Road, Newcastle upon Tyne NE4 6BE.

Correspondence: K. Snell.

Received 25 April 1983; accepted 22 June 1983.

Studies to date have largely failed to distinguish between these mechanisms as most have been concerned with the tumours themselves rather than with the early events of carcinogenesis. The work presented here has involved the histological and biochemical investigation of both early and late stages of hepatocarcinogenesis in relation to normal hepatocyte differentiation, as well as the changes occurring during liver regeneration following partial hepatectomy. During normal liver development a number of enzymes undergo marked changes in their activity at certain physiologically critical times: late foetal, neonatal and weaning stages (Greengard, 1971; Snell, 1981). Nine enzymes which show such developmental phase-specific profiles of activity and which were selected entirely on the basis of their serving as reliable markers of each critical phase of differentiation were studied during the course of diethylnitrosamine-induced hepatocarcinogenesis and during liver regeneration after partial hepatectomy. For comparison the activity profiles of these enzymes during normal liver development were also determined. A number of considerations governed the selection of the various enzymes used as differentiation markers in the present study. It is important to include enzymes that increase in amount during biochemical dedifferentiation (foetal enzymes) as well as those that decrease (adult enzymes) in these circumstances. This avoids reliance on the loss of enzyme activities that could well result from non-specific effects related to the toxicity of the tumour-inducing chemical or related to non-neoplastic pathological events occurring in the tissue as a result of tumour growth. Again, by selecting enzymes from different pathways of metabolism as indicators of differentiation there is less chance of a non-neoplastic change, such as alterations in nutritional or hormonal status, leading to a

© The Macmillan Press Ltd., 1983

concerted effect on all enzymes if they serve different metabolic functions. With a range of developmental enzyme indicators selected by these criteria, it is unlikely that any non-specific physiological or pathological factor would result in changes in all the enzymes in the direction characteristic of the dedifferentiated state. Diethylnitrosamine was the carcinogen used in this study because of its high carcinogenicity and low toxicity (Magee & Barnes, 1967).

### Materials and methods

Wistar Albino rats (from the breeding colony of the Animal Unit, University of Surrey) were used for all experiments, except the transplantable hepatoma studies. Male animals were used for all experiments except for those on foetal animals (sex not determined). Two transplantable hepatomas, one rapidly growing (3 weeks between transplantations) designated UA, and one more slowly growing (4 weeks between transplantations) designated WDA, originally induced by ethionine (Reid, 1970), were passaged by subcutaneous implantation into the flanks of male Chester Beatty hooded rats (from a breeding colony at the Animal Unit, University of Surrey). All animals were allowed food (Laboratory diet 1, Spratts Patent Ltd., Barking, London, UK) and water *ad libitum*.

Diethylnitrosamine (DEN) (Eastman Kodak Co., Kirby, Liverpool, UK) was administered in the drinking water to rats weighing 100–120 g at the beginning of the experiment. DEN-treated animals gained weight over the course of the experiment and at any stage were at least 85% of the weight of age-matched controls. In control animals such weight differences had a negligible effect on any of the enzyme activities measured. The concentration of DEN was adjusted weekly so that the animals received 10 mg DEN $kg^{-1}$ body wt per day. This particular regime gives rise to hepatocellular carcinomas with a mean induction time of 14 weeks (Druckrey, 1967; N.J. Curtin, unpublished observations). Animals were killed at 2, 4, 6, 8, 10 and 11 weeks, and in a preliminary experiment at 15 weeks. Partial hepatectomies (65–70%) were performed on adult rats in the weight range 150–250 g under ether anaesthesia by the technique of Higgins & Anderson (1931). Animals were killed 18 h, 24 h, 48 h, 3 d, 5 d and 7 d after operation.

For hepatic enzyme assays L-malate, glucose 6-phosphate, ATP, IDP and thymidine were obtained from Sigma Ltd. (Poole, Dorset, UK). 2-oxoglutarate, phosphoenolpyruvate, NADH, glucose 6-phosphate dehydrogenase (from yeast) and malate dehydrogenase (from pig heart) were obtained from Boehringer Corporation (Lewes, E. Sussex, UK). NADP was purchased from Cambrian Chemicals (Croydon, Surrey, UK) and $NaH^{14}CO_3$ (50 $\mu$Ci $ml^{-1}$; 0.1 mCi $mmol^{-1}$) and 2-$^{14}$C thymidine (50 $\mu$Ci $ml^{-1}$; 56.7 mCi $mmol^{-1}$) were obtained from the Radiochemical Centre (Amersham, Bucks., UK). 5 g of 2,5-diphenyloxyazole (PPO) and 0.5 g of 1,4-bis 2-(5-phenoxyozole)-benzene (POPOP) from Packard Instruments Inc. (Downes Grove, Ill., USA) were dissolved in 1 l of toluene as scintillant and for counting aqueous samples they were dissolved in 667 ml of toluene made up to 1 l with Metapol or Synperonic NX detergent (Durham Chemical Distribution, Birtley, Tyne & Wear, UK). All remaining chemicals were of Analar grade from BDH (Poole, Dorset, UK).

Liver or hepatoma tissue for hepatic enzyme assays was homogenised in 2 vol of ice-cold 0.15 M KCl–0.2 mM $KHCO_3$. Aliquots were diluted with homogenising medium to give a 10% homogenate, some of which was then further diluted with distilled water to give a 1% homogenate. The 1% homogenate was sonicated for 3 × 10 seconds with a Soniprobe Type 1130A (Daw Instruments Ltd., London, UK). The remaining 33% homogenate was centrifuged for 1 h at 100,000 *g* at 4°C in an MSE Superspeed 50 centrifuge. Spectrophotometric assays were followed using a Gilford 250 spectrophotometer. Scintillation counting was carried out in an LKB-Wallac 1210.

Glucose 6-phosphatase (EC 3.1.3.9) was assayed at 37°C in the 10% homogenate using 25 mM tris-maleate buffer, pH 6.7, by the method of Nordlie & Arion (1966). Glutamate dehydrogenase (EC 1.4.1.2) (Herzfeld, 1972) and aspartate aminotransferase (EC 2.6.1.1) (Herzfeld & Greengard, 1971) were assayed in the 1% sonicated homogenate at 30°C. Thymidine kinase (EC 2.7.1.21) was assayed in the 100,000 *g* supernatant at 37°C (Weber *et al.*, 1978) using Tris-HCl buffer pH 8.0, and phosphoenolpyruvate carboxykinase (EC 4.1.1.32) at 30°C (Ballard & Hanson, 1967). Glucokinase (EC 2.7.1.2) and hexokinase (2.7.1.1) were assayed in parallel in the 100,000 *g* supernatant at 30°C (Sharma *et al.*, 1963). Malic enzyme (EC 1.1.1.40) (Hsu & Lardy, 1969) and glucose 6-phosphate dehydrogenase (EC 1.1.1.44) (Langdon, 1966) were assayed at 30°C in the 100,000 *g* supernatant.

### Results

### *Enzyme changes in differentiating and regenerating liver and during hepatocarcinogenesis*

The values obtained for liver enzyme activities during development are given in Table I together with their designated developmental clusters. TK and HK activities decreased during development,

AAT, GDH and G6Pase first appeared in the foetal period and continued to increase after birth with adult values achieved at weaning. PEPCK rose sharply at birth, then gradually decreased to adult values on weaning. GK and malic enzyme appeared at weaning. The developmental profiles are in general agreement with previous studies (see Greengard, 1971; Knox, 1976; Snell, 1981). Changes in enzyme activities during the course of DEN-induced hepatocarcinogenesis are shown in Table II. From the time of the first observation (at 2 weeks) HK and TK were elevated and all other enzymes were decreased. G6PDH later increased above control values so that all three "high foetal cluster" enzymes were elevated, and enzymes belonging to more differentiated clusters were decreased. However malic enzyme, belonging to the "weaning cluster", was increased or unchanged at the later stages of DEN treatment. Comparison of Tables I and II suggests that whereas some of the changes in enzyme activities during carcinogenesis are similar to what would be expected from a step-wise reversal of differentiation (*viz.* the pattern of changes in TK, HK, G6PDH and GK), the changes in activities of the other enzymes did not follow a strict course of developmental reversal. Nevertheless, if Spearman's Rank Correlation analysis is applied to all the data a general pattern does begin to emerge (Table III). The principles and applications of this statistical analysis are explained more fully elsewhere (Knox, 1976). Briefly, the activities of the enzymes normalized to liver units (by comparison with values obtained for control adult animals; where activity in adult liver is taken as 1.0 liver unit, as recommended by Knox, 1976) are ranked for each set of observations (developmental age or time of DEN treatment). The rank correlation coefficient $R_s$ and Student's $t$-value being calculated as follows:

$$R_s = 1 - \frac{6d^2}{(n-1)n(n+1)}$$

$$t = R_s \sqrt{\frac{n-2}{1-R_s^2}}$$

where $n-2$ are the number of degrees of freedom, $d$ is the difference in rank of each observation between the two sets and $n$ is the number of observations. This enables the enzymic pattern in two different states to be compared.

After 2 weeks of DEN treatment there was no significant correlation with any stage of development, however at 4 weeks the liver enzyme pattern of the treated animals showed a significant correlation with both the weanling animals ($P<0.01$) and the 5-day-old animals ($P<0.1$). From 6 weeks of DEN treatment onwards the only significant correlation was with foetal liver ($P<0.05$). In the early stages, up to 4 weeks of treatment with DEN, histological changes were found to be minimal (N.J. Curtin & K. Snell, in preparation), and this suggests that the enzymic changes are unlikely to be due to any toxic effects of DEN. After 6 weeks of treatment more marked morphological changes were apparent (viz., fibrosis and hyperplasia), although the liver was not classified as preneoplastic on histological grounds at this stage (N.J. Curtin & K. Snell, in preparation). The appearance of nodular preneoplastic foci after 10 weeks of DEN treatment was associated with enzyme changes that were exaggerated in the nodules (as assessed histochemically) but were not confined to these focal areas. The enzymic pattern of primary tumours arising at 15 weeks in this study showed a significant correlation with the livers of animals treated for 11 weeks with DEN alone ($P<0.01$), and with the WDA transplantable hepatoma ($P<0.01$) and the UA transplantable hepatoma ($P<0.05$) (results not shown in detail, see Table VI). There was also a significant correlation of enzyme patterns of both transplantable hepatomas with foetal livers $P<0.05$ and with rats treated for 11 weeks with DEN ($P<0.01$ or better) (Table VI).

Changes in enzyme activities during liver regeneration after partial hepatectomy are shown in Table IV. Spearman's Rank Correlation analysis (Table V) shows that at 18 h after hepatectomy there was a significant correlation with the enzymic pattern of the weanling rat liver ($P<0.05$); at 24 h there was a correlation with the 5- and 10-day-old rat liver ($P<0.05$); at 48 h the hepatectomised liver showed significant correlations with all stages of development up to 15 days ($P<0.05$). At 3 days after hepatectomy the only correlation was with foetal rat liver ($P<0.005$); at 5 days no correlations were evident; and at 7 days there was a significant correlation with the 5-day-old rat liver ($P<0.05$). Thus it seems that sequential changes in liver enzymes assume first a postnatal pattern, then a foetal pattern and then a postnatal pattern again, i.e. that there is a retrodifferentiation followed by a redifferentiation. When enzyme profiles in the hepatectomised rats at 18 and 24 h were compared with DEN-treated rats, the only correlation was seen with the early stages of carcinogenesis at 4 ($P<0.005$) and 2 ($P<0.05$) weeks of DEN treatment respectively. At 48 h after hepatectomy there was a correlation with both early stages (2 weeks, $P<0.01$) and late stages (11 weeks, $P<0.05$) of DEN treatment. At 3 days after hepatectomy there was a correlation with all stages of hepato-carcinogenesis, the most marked being with 10 and 11 weeks of DEN treatment ($P<0.005$). After 3 days post-hepatectomy the correlation with the

Statistical significance is given by: $^{+}$, $P<0.1$; *, $P<0.05$; **, $P<0.01$; ***, $P<0.005$.

**Table VI** Spearman's rank correlation analysis of the enzyme patterns of transplantable hepatomas and host liver compared with the enzyme patterns of liver during development, regeneration and primary hepatocarcinogenesis

| Compared tissue | *UA tumour* | | *WDA tumour* | |
|---|---|---|---|---|
| | *Rs* | *t* | *Rs* | *t* |
| Foetal | 0.7042 | 2.624* | 0.7292 | 2.819* |
| Newborn | 0.5272 | 1.65 | 0.3125 | 0.870 |
| 5 day old | 0.5458 | 1.724 | 0.3167 | 0.883 |
| 10 day old | 0.3708 | 1.056 | 0.0667 | 0.177 |
| 15 day old | 0.3542 | 1.002 | 0.2 | 0.54 |
| Weanling | 0.5542 | 1.761 | 0.1167 | 0.311 |
| DEN 11 weeks | 0.7875 | 3.381** | 0.9833 | 14.31*** |
| Host liver in DEN rats | −0.0208 | −0.055 | −0.1833 | −0.493 |
| Tumours in DEN rats | 0.7542 | 3.039* | 0.8167 | 3.744** |
| Regenerating liver | | | | |
| 18 h p.h. | 0.8292 | 3.924** | 0.6167 | 0.2073$^{+}$ |
| 24 h p.h. | 0.6625 | 2.34$^{+}$ | 0.2833 | 0.782 |
| 48 h p.h. | 0.8958 | 5.333*** | 0.6833 | 2.476* |
| 3 day p.h. | 0.9208 | 6.248*** | 0.95 | 8.05*** |
| 5 day p.h. | 0.8458 | 4.195** | 0.7125 | 2.687* |
| 7 day p.h. | 0.7875 | 3.381* | 0.5833 | 1.9$^{+}$ |

Statistical significance is given by: $^{+}$, $P<0.1$; *, $P<0.05$; **, $P<0.01$; ***, $P<0.005$.

later stages of hepatocarcinogenesis was no longer evident while that with early stages remained. There was a significant correlation of the 3-day post-hepatectomy liver with both the rapidly growing UA transplantable hepatoma ($P<0.005$) and the slow growing WDA transplantable hepatoma ($P<0.005$) (Table VI).

### Discussion

The enzymic changes observed during the course of chronic DEN-induced carcinogenesis and during liver regeneration are consistent with the view that these conditions are accompanied by a progressive return to a foetal enzymic pattern. This adds weight to the hypothesis of Uriel (1976) that the development of neoplasia is accompanied by a step-wise retrodifferentiation of adult cells to immature cells. However, the conclusions of our study are not in agreement with those of Malkin *et al.* (1978) who stated that there was no evidence for a step-wise retrodifferentiation or developmental phase-specific block in the enzymic pattern of either transplantable hepatomas or primary tumours induced by DEN plus acetamidofluorene. However these workers were inconsistent in their choice of which particular developmental stage (varying from early foetal to 4 weeks *post partum*) they used to represent the enzyme activities characteristic of immature liver.

The changes in enzyme activity occurred before marked histological changes were detected and are unlikely to be a toxic response to DEN. Moreover the direction of the enzyme changes was the same as that observed focally at later stages of carcinogenesis in preneoplastic nodules and later still in the primary tumours which developed. This persistence of the *nature* of the enzyme changes throughout carcinogenesis and in the final tumours suggests that the changes are related to the neoplastic process *per se* rather than being a feature of a generalised toxic response. In the absence of hepatotoxic agents which are unequivocally known not to be carcinogenic, it was not possible to investigate the effects of hepatotoxicity directly on enzyme activities. Nevertheless the fact that detectable changes are found at an early stage of carcinogenesis implies that this is a general hepatocellular response which only assumes a more focal nature as carcinogenesis progresses and as particular groups of cells become "fixed" in the neoplastic state. Since the enzyme changes appear to precede any detectable histological features characteristic of neoplasia, there is the prospect that the development of such an enzymic pattern may be useful as an indicator of *in vivo* carcinogenesis in toxicological screening trials, instead of waiting for the development of frank tumours, thereby significantly shortening the duration of such trials.

Of the changes observed in regenerating liver the elevation of PEPCK and decrease in GK activities have been reported previously (Katz, 1979). These enzyme adaptations contribute to the metabolic changes which subserve the need for greater gluconeogenesis by the small portion of liver remaining. These authors did not report a decrease in G6Pase and increase in HK (which would tend to counteract the enhanced gluconeogenesis) as observed in the present work. At 3 days after partial hepatectomy PEPCK activity was lower than normal (as were the activities of the other postnatal enzymes) and it was at this time that a correlation with foetal and neoplastic enzymic patterns was observed. G6PDH, a key enzyme of the pentose phosphate pathway, whilst being elevated in the pre-replicative phase (18 h post-hepatectomy) was reduced during the period of most rapid growth. This finding was unexpected as the pentose phosphate pathway, which generates NADPH and ribose, is thought to be integral to DNA synthesis. Longenecker & Williams (1979) also observed that pentose phosphate pathway activity was reduced in liver cells after partial hepatectomy and suggest that either there is a large increase in glucose metabolism during regeneration which ensures sufficient carbon flux through the pentose phosphate pathway to support the synthesis of the required nucleic acid precursors, or that there is no increase and our view of the role of this pathway must be re-evaluated.

In our study we found that regenerating liver assumes an enzymic pattern similar to both (pre-) neoplastic liver and foetal and immature liver, although contrary views have been expressed (see Weber, 1975; Knox, 1976). Most previous studies have centered on regenerating liver at 24 h after partial hepatectomy at which time there is an underlying synchrony of cell division with most cells being in the pre-replicative phase and only a few mitotically active. Much of the liver growth at this time is due to cellular hypertrophy. The results reported here confirm that the liver 24 h after partial hepatectomy is different from foetal and/or (pre-)neoplastic liver, but that at 3 days post hepatectomy the regenerating liver bears a strong enzymic resemblance to foetal and neoplastic liver.

Taken altogether the present results suggest that an underlying retrodifferentiation process is common to both the process of hepatocarcinogenesis and liver regeneration. Although it is possible that persistent low grade DEN toxicity could be partly responsible for changes that might induce the liver to assume a dedifferentiated enzymic pattern similar to that of regenerating liver, the fact that the carcinogen treated liver does not revert to a normal pattern, as in the case of the regenerating liver, argues that other changes associated with DEN and related to its carcinogenicity are responsible for the observed persistent enzymic dedifferentiation. The working hypothesis is that whereas preneoplastic hepatocytes remain trapped in the foetal state (possibly as a result of the promotion phase of carcinogenesis), the regenerating hepatocytes retain the capacity to redifferentiate along the normal pathway of liver development. This interpretation is consistent with a view expressed previously (Walker & Potter, 1972) that a fully mature hepatocyte cannot undergo division without some degree of dedifferentiation, and that similar stages of dedifferentiation occur in both regenerating and precancerous liver. Similarly Uriel (1979) maintains that tissue regeneration and neoplastic change are both accompanied by the recapitulation of ontogeny in a reverse sequence. In the case of regeneration, as tissue renewal is accomplished there is a redifferentiation along the same pathway to give rise to cells showing similar characteristics to the original cell population.

Although the biochemical resemblance of neoplastic cells to foetal cells is significant it is by no means an exclusive feature, as similar characteristics can be seen in non-cancerous regenerating cells. Thus, certain "biochemical markers" of neoplasia, such as increases in $\alpha$-foetoprotein, $\gamma$-glutamyltranspeptidase, G6PDH and foetal isoenzymes and decreases in G6Pase and adult isoenzymes, may also be found in regenerating liver following surgery or toxic injury (Ideo *et al.*, 1971; Harada *et al.*, 1976; Taketa *et al.*, 1976; Stillman & Sell, 1979). Since retrodifferentiation is apparently a process underlying both neoplastic development and regeneration, the discovery of markers exclusive to neoplasia appears to be unlikely. However, the elevation of PEPCK at most stages of regeneration and its decrease during the course of carcinogenesis could possibly be used as a discriminant between malignant and non-malignant cell replication. Furthermore our study emphasises that it may be possible to distinguish between malignant and non-malignant hyperplasia on the basis of quantitative, rather than qualitative, differences between these two states; for, although the pattern of enzyme activities is similar, the magnitude of change is greater in the (pre-)neoplastic liver (despite a lesser degree of hyperplasia and proportion of liver tissue involved) than in the regenerating liver. A further degree of resolution might also be achieved by investigating the levels of isoenzymes of developmental-phase specific enzymes rather than total enzyme activity. In the present work this was not investigated systematically, although the measurements of hexokinase and glucokinase in fact distinguish between two groups of glucose phosphorylating isoenzymes. Since isoenzymes are by definition the protein products of different independent genes, their differential expression during normal liver development and during the course of hepatocarcinogenesis could provide more precise indications of modifications in gene expression that might be common to the two situations.

We are grateful to the Cancer Research Campaign and the Science and Engineering Research Council for financial support. We also thank Prof. W.E. Knox (Harvard Medical School) for introducing us to the use of Rank Correlation analysis.

### References

ABELEV, G.I. (1971). Alpha-fetoprotein in ontogenesis and its association with malignant tumours. *Adv. Cancer Res.*, **14**, 295.

ANDERSON, N.G. & COGGIN, J.H. (1974). The interrelations between development, retrogenesis, viral transformation and human cancer. In: *The Cell Surface and Development* (ed. Moscana), New York: Wiley Interscience, p. 297.

BALLARD, F.J. & HANSON, R.W. (1967). Phosphoenolpyruvate carboxykinase and pyruvate carboxylase in developing rat liver. *Biochem. J.*, **104**, 866.

CRISS, W.E. (1971). A review of isozymes in cancer. *Cancer Res.*, **31**, 1523.

DRUCKREY, H. (1967). Quantitative aspects in chemical carcinogenesis. *IUCC Monogr. Series*, **7**, 60.

ENOMOTO, K., DEMPO, K., MORI, M. & ONOE, T. (1978). Histopathological and ultrastructural study on extramedullary hematopoietic foci in early stage of 3′-methyl-4-(dimethylamino)azobenzene hepatocarcinogenesis. *Gann*, **69**, 249.

FARBER, E. (1976). The pathology of experimental liver cell cancer. In: *Liver Cell Cancer* (eds. Cameron *et al.*) Amsterdam: Elsevier, p. 243.

FISHMAN, W.H. & SINGER, R.M. (1975). Ectopic isoenzymes: Expression of embryonic genes in neoplasia. In: *Cancer: A Comprehensive Treatise* (ed. Becker), Vol. 3, p. 57. New York: Plenum Press.

GREENGARD, O. (1971). Enzymic differentiation in mammalian tissues. *Essays Biochem.*, **7**, 159.

GREENSTEIN, J.P. (1954). *The Biochemistry of Cancer.* New York: Academic Press.

HARADA, M., OKABE, K., SHIBATA, K., MASUDA, H., MIYATA, K. & ENOMOTO, M. (1976). Histochemical demonstration of increased gamma-glutamyl transpeptidase in rat liver during hepatocarcinogenesis. *Acta Histochem. Cytochem.*, **9**, 168.

HERZFELD, A. (1972). The distribution of glutamate dehydrogenase in rat tissues. *Enzyme*, **13**, 246.

HERZFELD, A. & GREENGARD, O. (1971). Aspartate aminotransferase in rat tissues: Changes with growth and hormones. *Biochim. Biophys. Acta*, **237**, 88.

HIGGINS, G.M. & ANDERSON, R.M. (1931). Experimental pathology of the liver. 1. Restoration of the liver of the white rat following partial surgical removal. *Arch. Pathol.*, **12**, 186.

HSU, R.Y. & LARDY, H.A. (1969). Malic enzyme. *Methods Enzymol.*, **13**, 230.

ICHIHARA, A. (1975). Isozyme pattern of branched chain amino acid transaminase during cellular differentiation and carcinogenesis. *Ann. N.Y. Acad. Sci.*, **259**, 347.

IDEO, G., DEL NINNO, E. & DE FRANCHIS, R. (1977). Behaviour of some enzymes and isoenzymes in plasma, liver and bile of rats treated with $CCl_4$. *Enzyme*, **12**, 242.

KATZ, N. (1979). Correlation between rates and enzyme levels of increased gluconeogenesis in rat liver and kidney after partial hepatectomy. *Eur. J. Biochem.*, **98**, 535.

KNOX, W.E. (1976). *Enzyme Patterns in Fetal, Adult and Neoplastic Rat Tissues.* 2nd edn. Basel: S. Karger.

LANGDON, R.G. (1966). Glucose 6-phosphate dehydrogenase from erythrocytes. *Methods Enzymol.*, **9**, 126.

LONGENECKER, J.P. & WILLIAMS, J.F. (1979). Pentose phosphate cycle in regenerating liver. *Proc. Aust. Biochem. Soc.*, **12**, 53.

MAGEE, P.N. & BARNES, J.M. (1967) *Adv. Cancer Res.*, **10**, 163.

MALKIN, A., KELLER, J.A. & CAPLAN, P. (1978). Phase specific profiles of hepatic enzyme activity during ontogeny and experimental hepatomas in rats. *Scand. J. Immunol. Suppl.*, **8**, 447.

NORDLIE, R.C. & ARION, W.J. (1966). Glucose 6-phosphatase. *Methods Enzymol.*, **9**, 619.

PIERCE, G.B. (1970). Differentiation of normal and malignant cells. *Fed. Proc.*, **29**, 1248.

REID, E. (1970). Specificity of certain biochemical derangements in hepatic carcinogenesis. *Br. J. Cancer*, **24**, 128.

SCHAPIRA, F. (1973). Isozymes and cancer. *Adv. Cancer Res.*, **18**, 77.

SHARMA, C., MANJESHWAR, K. & WEINHOUSE, S. (1963). Effects of diet and insulin on glucose-adenosine triphosphate phosphotransferases of rat liver. *J. Biol. Chem.*, **238**, 3840.

SNELL, K. (1981). Developmental enzyme pathology. In: *Developmental Toxicology* (ed. Snell). London: Croom–Helm, p. 299.

STILLMAN, D. & SELL, S. (1979). Models of chemical hepatocarcinogenesis and oncodevelopmental gene expression. *Methods Cancer Research*, **18**, 135.

TAKETA, K., WATANABE, A. & KOSAKA, K. (1976). Undifferentiated gene expression in liver injuries. In: *Onco-Developmental Gene Expression* (eds Fishman & Sell). New York: Academic Press, p. 219.

URIEL, J. (1976). Cancer, retrodifferentiation and the myth of Faust. *Cancer Res.*, **36**, 4269.

URIEL, J. (1979). Retrodifferentiation and the fetal patterns of gene expression in cancer. *Adv. Cancer Res.*, **29**, 127.

WALKER, P.R. & POTTER, V.R. (1972). Isozyme studies on adult, regenerating, precancerous and developing liver in relation to findings in hepatomas. *Adv. Enz. Reg.*, **10**, 339.

WEBER, G. (1975). Biochemical strategy of the regenerating liver cell. In: *Liver Regeneration after Experimental Injury* (eds Lesch & Reutter). New York: Stratton Intercontinental Medical Book Co., p. 103.

WEBER, G., SHIOTANI, T., KIZAKI, H., TZENC, D., WILLIAMS, J.C. & GLADSTONE, N. (1978). Biochemical strategy of the genome as expressed in regulation of pyrimidine metabolism. *Adv. Enz. Reg.*, **16**, 3.


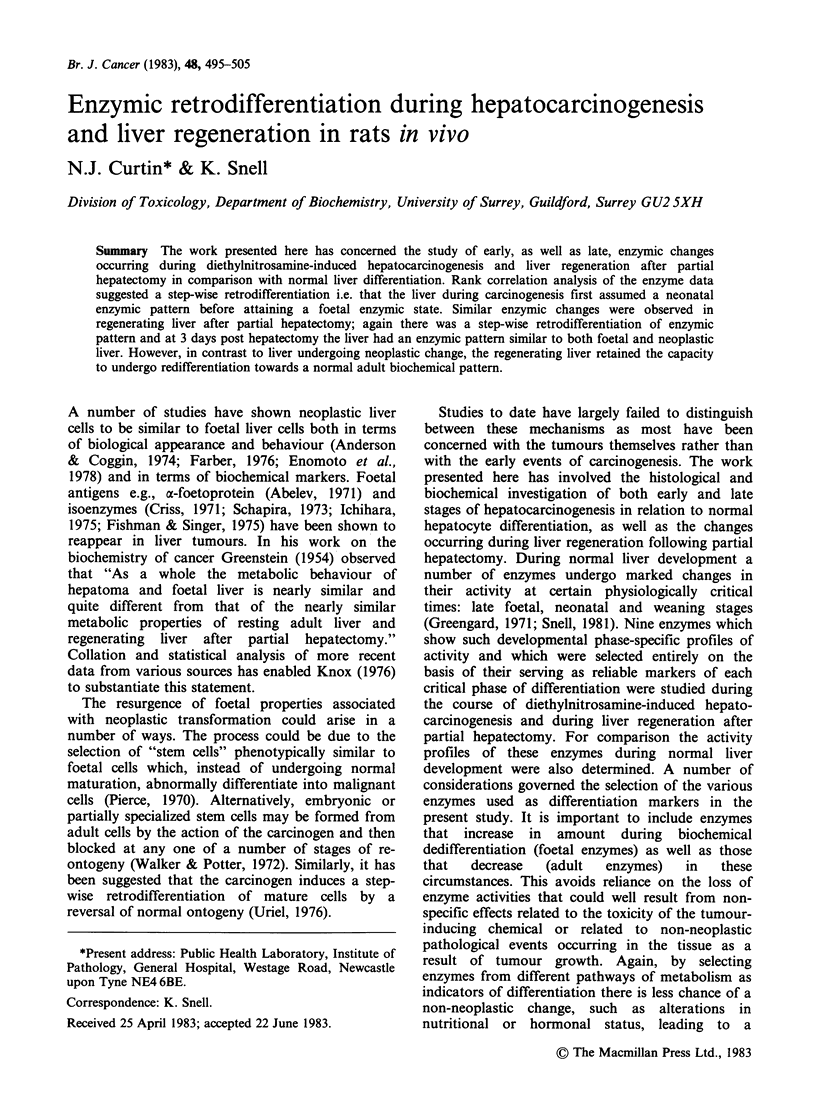

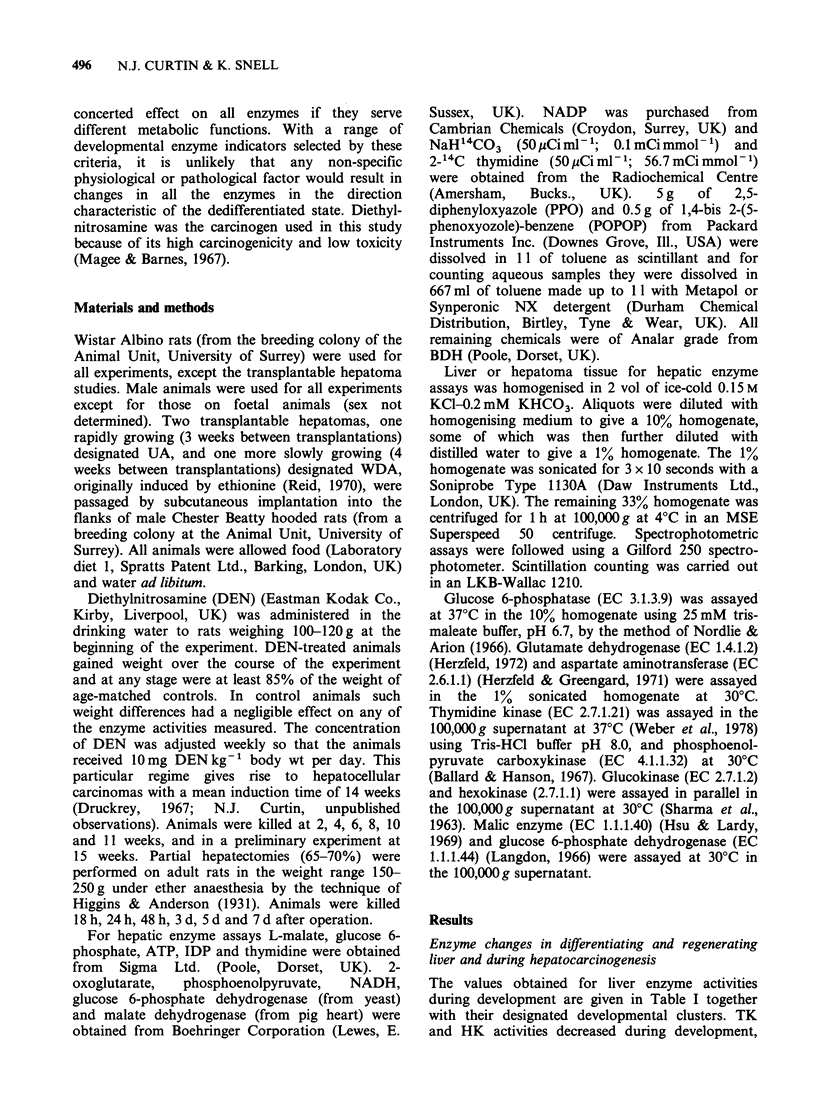

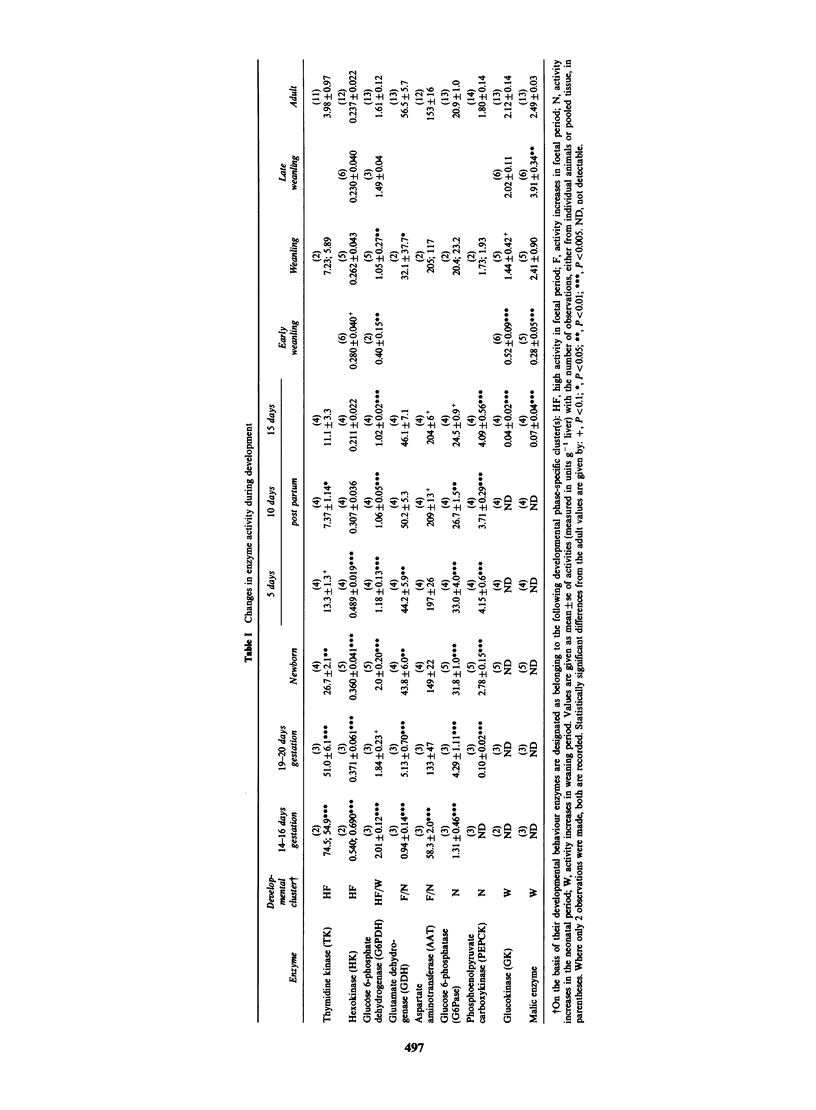

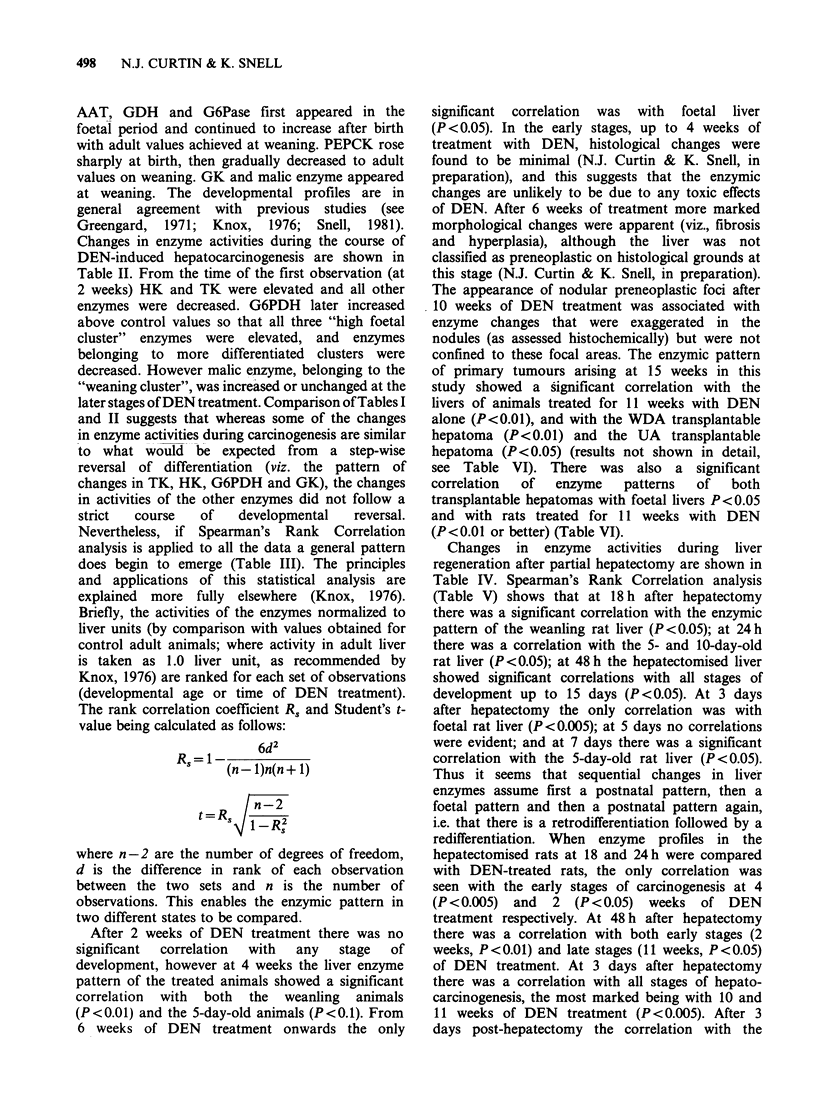

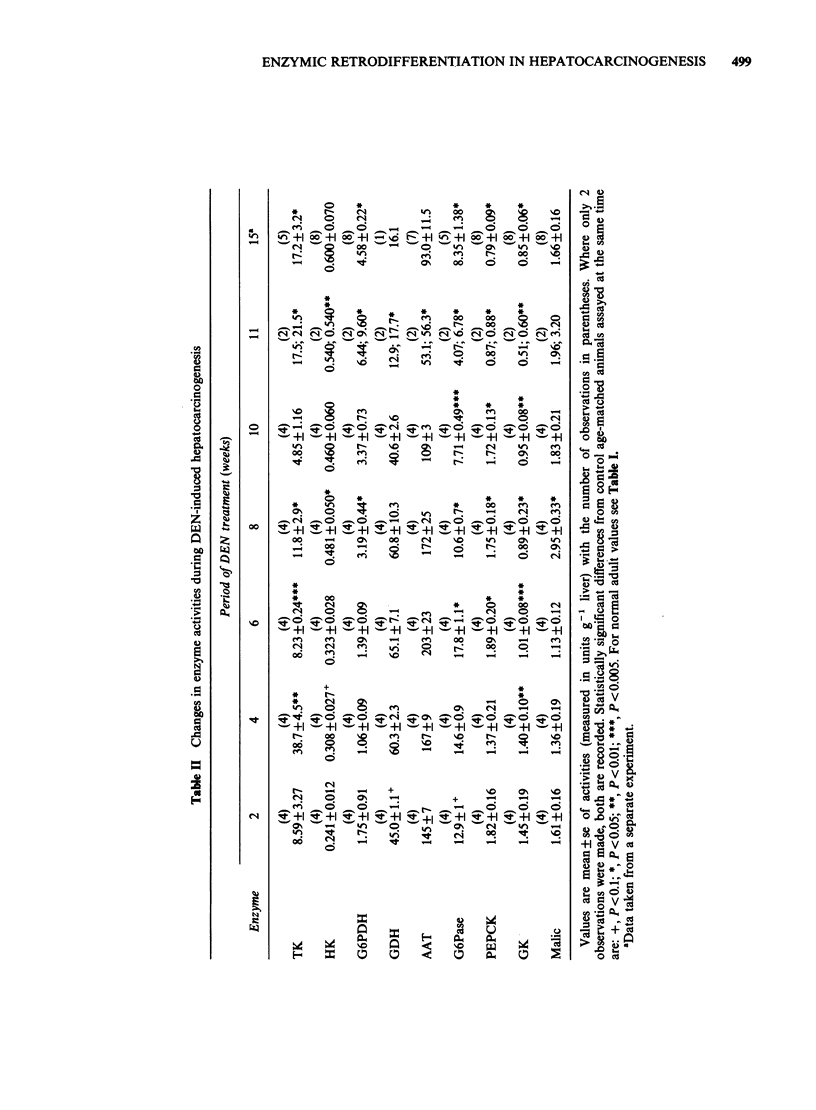

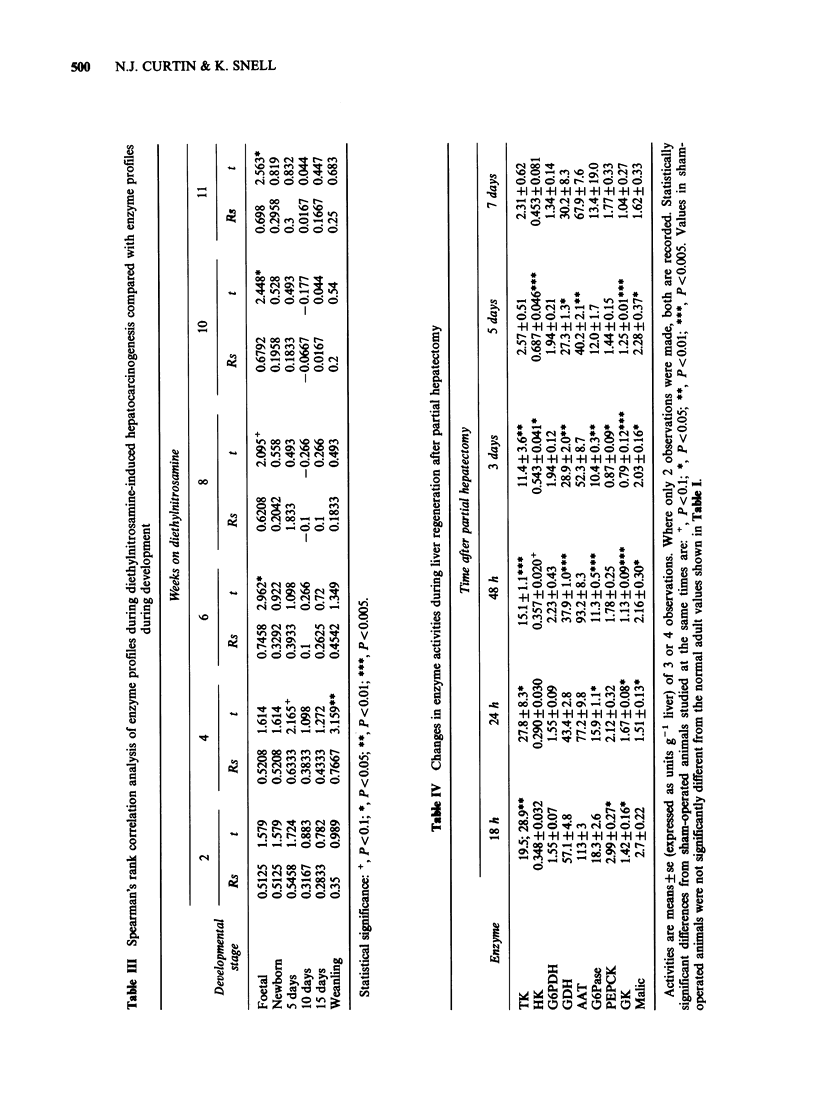

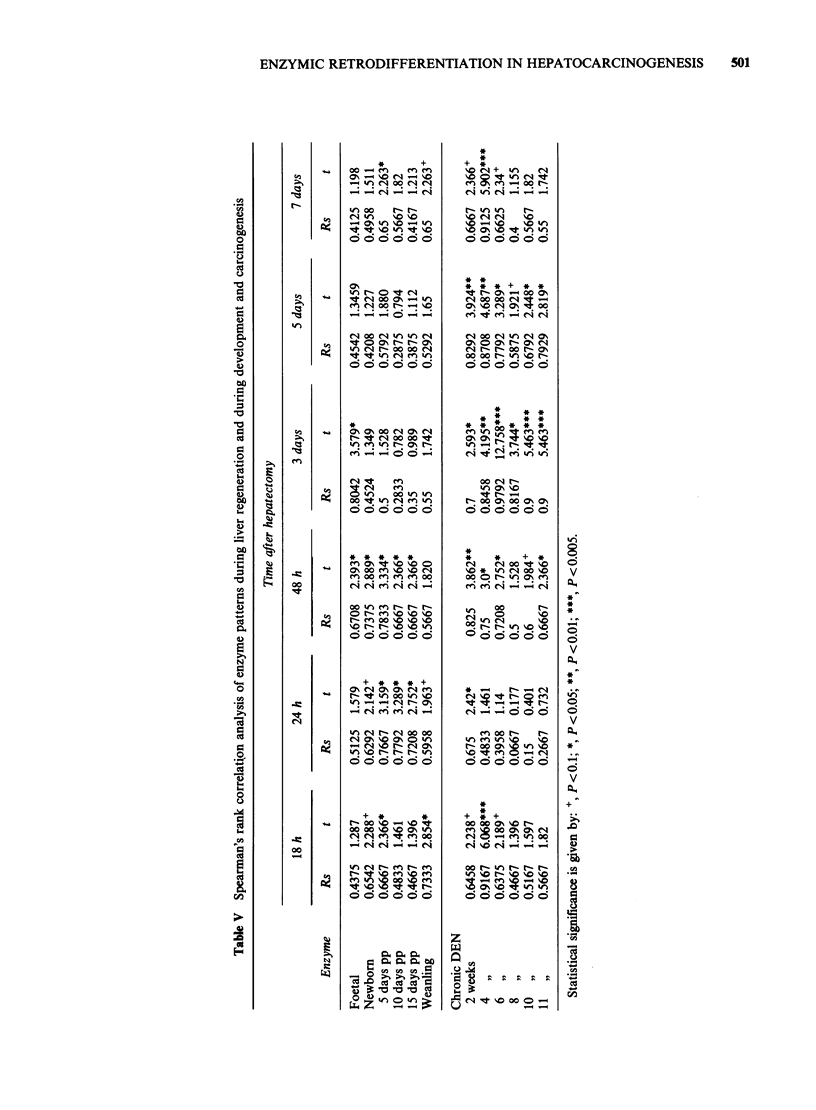

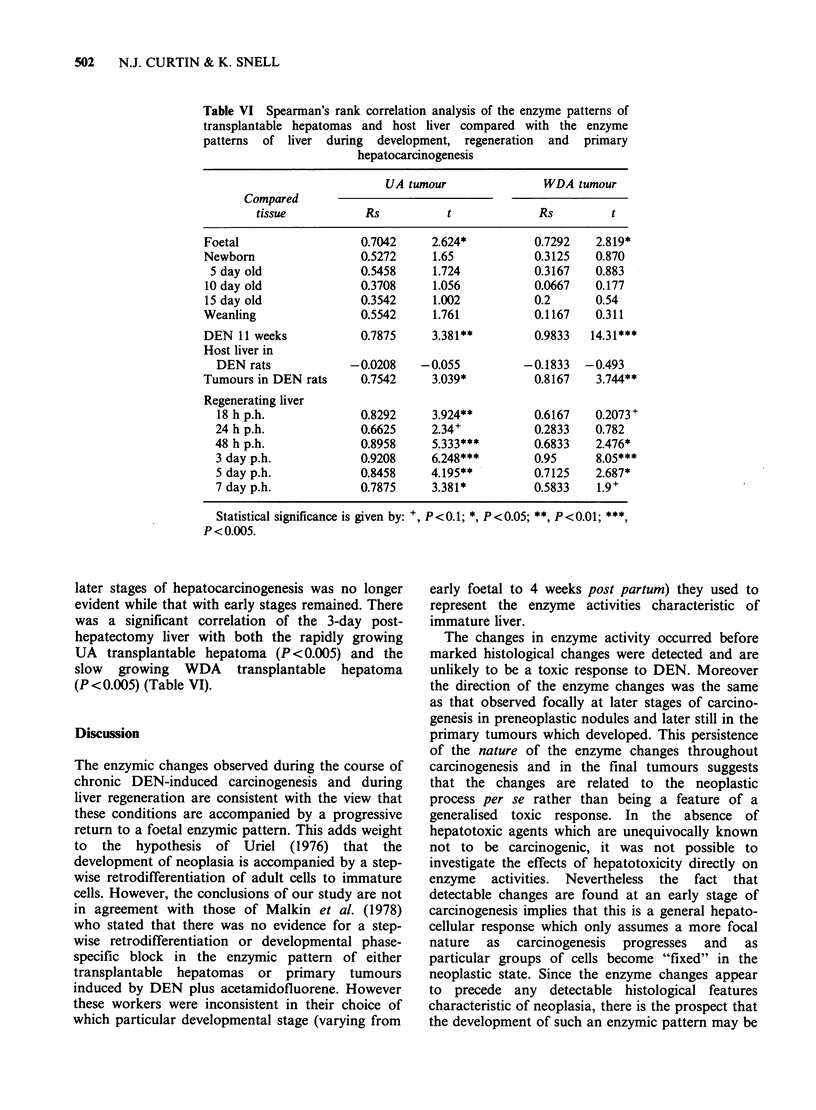

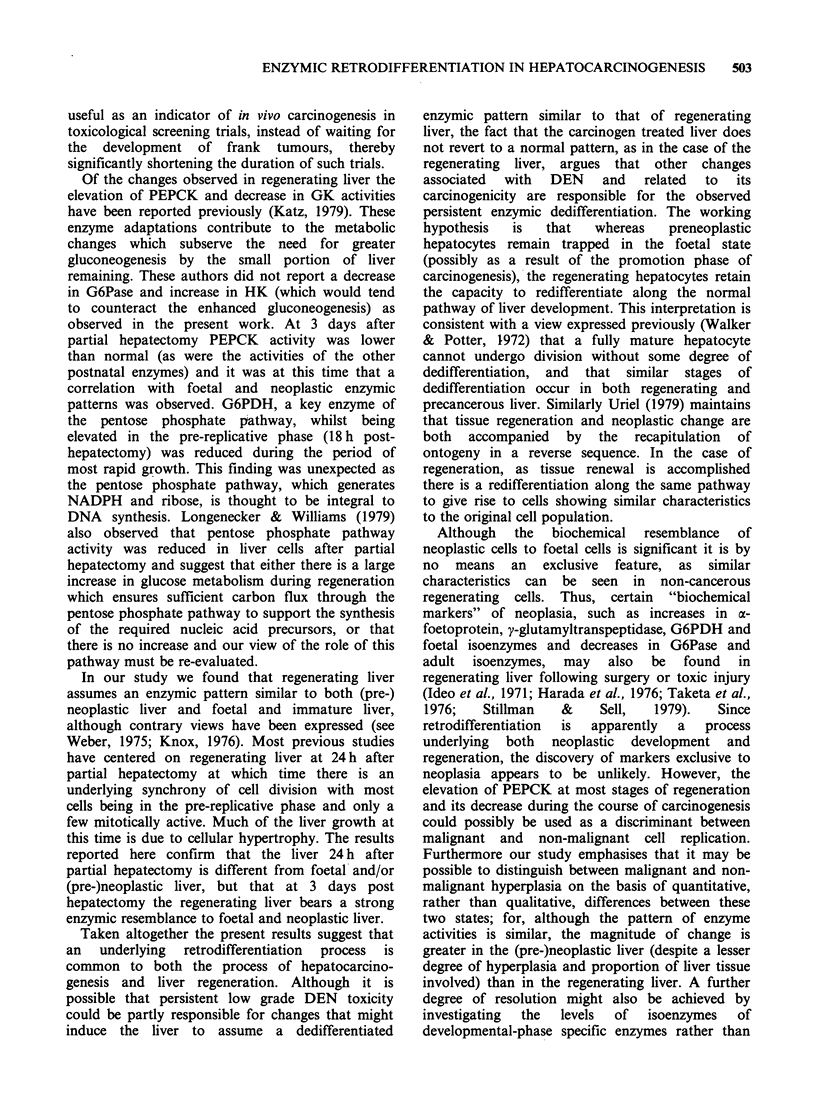

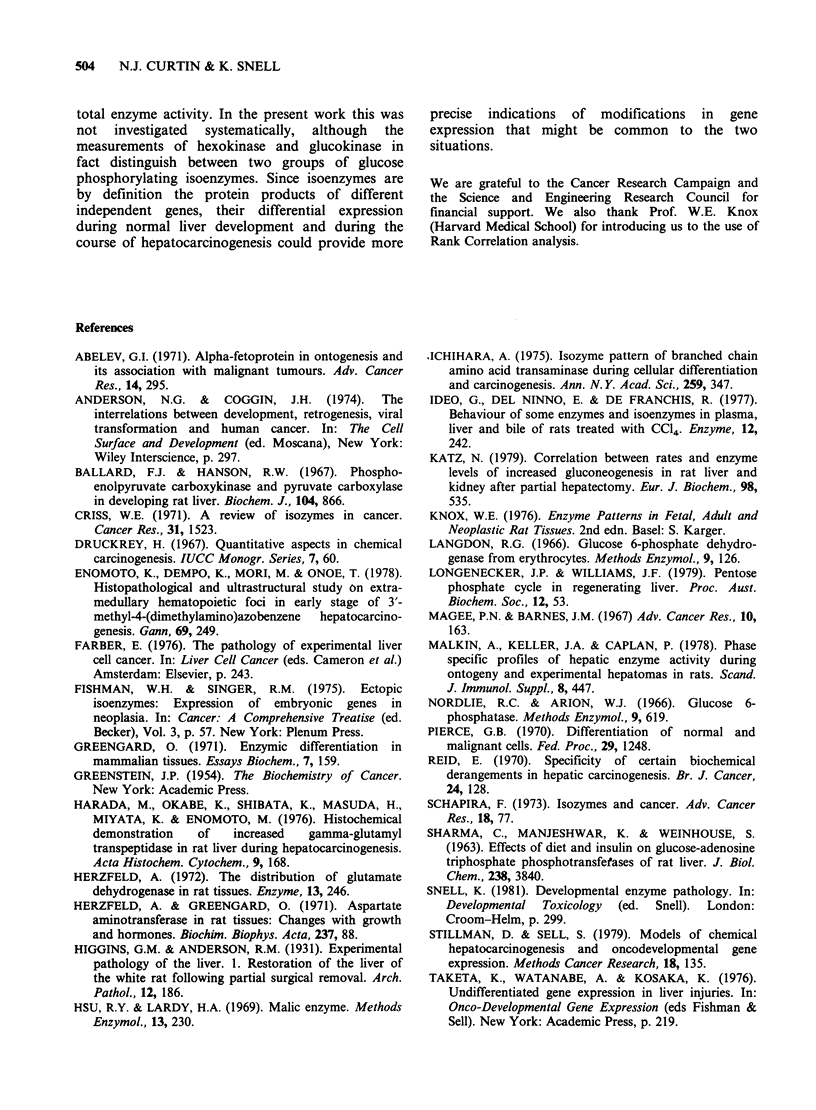

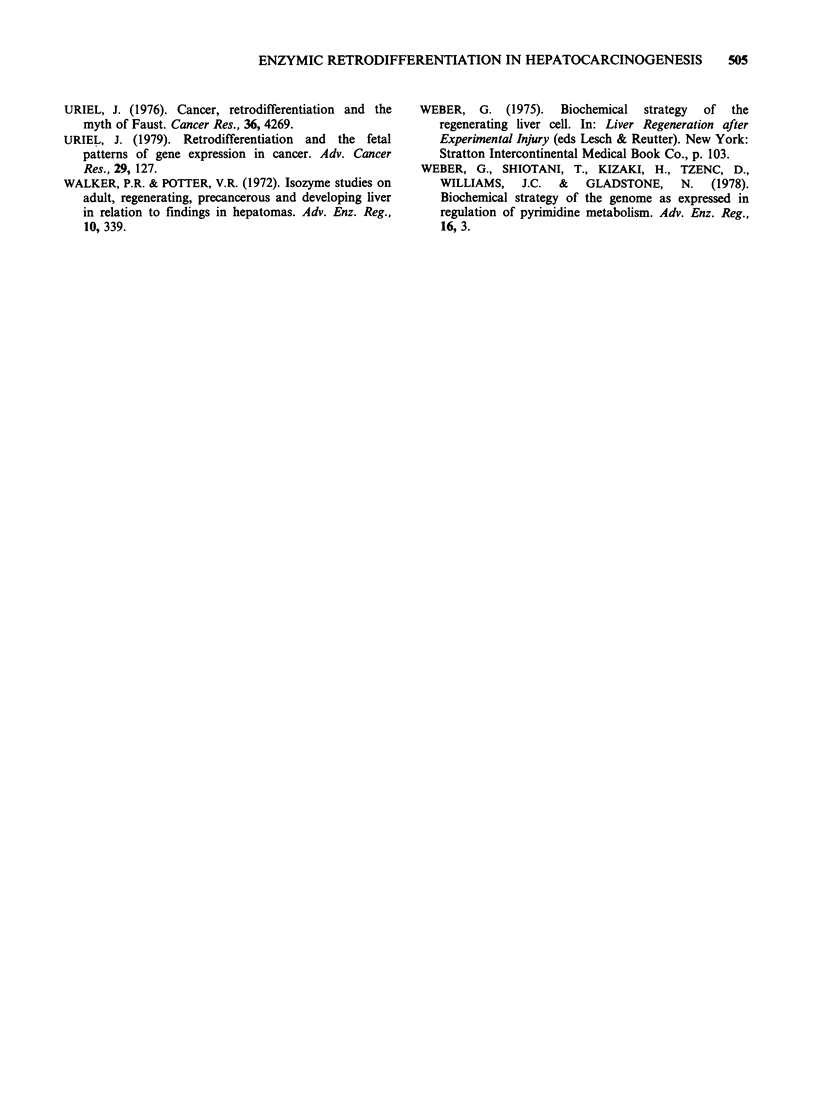


## References

[OCR_01242] Abelev G. I. (1971). Alpha-fetoprotein in ontogenesis and its association with malignant tumors.. Adv Cancer Res.

[OCR_01254] Ballard F. J., Hanson R. W. (1967). Phosphoenolpyruvate carboxykinase and pyruvate carboxylase in developing rat liver.. Biochem J.

[OCR_01259] Criss W. E. (1971). A review of isozymes in cancer.. Cancer Res.

[OCR_01267] Enomoto K., Dempo K., Mori M., Onoé T. (1978). Histopathological and ultrastructural study on extramedullary hematopoietic foci in early stage of 3'-methyl-4-(dimethylamino)azobenzene hepatocarcinogenesis.. Gan.

[OCR_01285] Greengard O. (1971). Enzymic differentiation in mammalian tissues.. Essays Biochem.

[OCR_01304] Herzfeld A., Greengard O. (1971). Aspartate aminotransferase in fat tissues: changes with growth and hormones.. Biochim Biophys Acta.

[OCR_01300] Herzfeld A. (1972). The distribution of glutamate dehydrogenase in rat tissues.. Enzyme.

[OCR_01329] Ichihara A. (1975). Isozyme patterns of branched-chain amino acid transaminase during cellular differentiation and carcinogenesis.. Ann N Y Acad Sci.

[OCR_01334] Idéo G., Del Ninno E., De Franchis R. (1971). Behaviour of some enzymes and isoenzymes in plasma liver and bile of rats treated with carbon tetrachloride.. Enzyme.

[OCR_01340] Katz N. (1979). Correlation between rates and enzyme levels of increased gluconeogenesis in rat liver and kidney after partial hepatectomy.. Eur J Biochem.

[OCR_01373] Pierce G. B. (1970). Differentiation of normal and malignant cells.. Fed Proc.

[OCR_01377] Reid E. (1970). Specificity of certain biochemical derangements in hepatocarcinogenesis.. Br J Cancer.

[OCR_01386] SHARMA C., MANJESHWAR R., WEINHOUSE S. (1963). EFFECTS OF DIET AND INSULIN ON GLUCOSE-ADENOSINE TRIPHOSPHATE PHOSPHOTRANSFERASES OF RAT LIVER.. J Biol Chem.

[OCR_01382] Schapira F. (1973). Isozymes and cancer.. Adv Cancer Res.

[OCR_01410] Uriel J. (1976). Cancer, retrodifferentiation, and the myth of Faust.. Cancer Res.

[OCR_01414] Uriel J. (1979). Retrodifferentiation and the fetal patterns of gene expression in cancer.. Adv Cancer Res.

[OCR_01419] Walker P. R., Potter V. R. (1972). Isozyme studies on adult, regenerating, precancerous and developing liver in relation to findings in hepatomas.. Adv Enzyme Regul.

[OCR_01431] Weber G., Shiotani T., Kizaki H., Tzeng D., Williams J. C., Gladstone N. (1977). Biochemical strategy of the genome as expressed in regulation of pyrimidine metabolism.. Adv Enzyme Regul.

